# Vitamin D Toxicity–A Clinical Perspective

**DOI:** 10.3389/fendo.2018.00550

**Published:** 2018-09-20

**Authors:** Ewa Marcinowska-Suchowierska, Małgorzata Kupisz-Urbańska, Jacek Łukaszkiewicz, Paweł Płudowski, Glenville Jones

**Affiliations:** ^1^Department of Geriatrics, Medical Centre for Postgraduate Education, Warsaw, Poland; ^2^Department of Biochemistry and Clinical Chemistry, Medical University of Warsaw, Warsaw, Poland; ^3^Department of Biochemistry, Radioimmunology and Experimental Medicine, Children's Memorial Health Institute, Warsaw, Poland; ^4^Department of Biomedical and Molecular Sciences, Queen's University, Kingston, ON, Canada

**Keywords:** vitamin D, 25(OH)D, toxicity, clinical symptoms, management

## Abstract

Confusion, apathy, recurrent vomiting, abdominal pain, polyuria, polydipsia, and dehydration are the most often noted clinical symptoms of vitamin D toxicity (VDT; also called vitamin D intoxication or hypervitaminosis D). VDT and its clinical manifestation, severe hypercalcemia, are related to excessive long-term intake of vitamin D, malfunctions of the vitamin D metabolic pathway, or the existence of coincident disease that produces the active vitamin D metabolite locally. Although VDT is rare, the health effects can be serious if it is not promptly identified. Many forms of exogenous (iatrogenic) and endogenous VDT exist. Exogenous VDT is usually caused by the inadvertent or improper intake of extremely high doses of pharmacological preparations of vitamin D and is associated with hypercalcemia. Serum 25-hydroxyvitamin D [25(OH)D] concentrations higher than 150 ng/ml (375 nmol/l) are the hallmark of VDT due to vitamin D overdosing. Endogenous VDT may develop from excessive production of an active vitamin D metabolite – 1,25(OH)_2_D in granulomatous disorders and in some lymphomas or from the reduced degradation of that metabolite in idiopathic infantile hypercalcemia. Endogenous VDT may also develop from an excessive production of 25(OH)D and 1,25(OH)_2_D in congenital disorders, such as Williams–Beuren syndrome. Laboratory testing during routine clinical examinations may reveal asymptomatic hypercalcemia caused by the intake of vitamin D even in doses recommended for the general population and considered safe. That phenomenon, called hypersensitivity to vitamin D, reflects dysregulated vitamin D metabolism. Researchers have proposed many processes to explain VDT. Those processes include elevated activity of 1α-hydroxylase or inhibited activity of 24-hydroxylase, both leading to increased concentration of 1,25(OH)D; increased number of vitamin D receptors; and saturation of the capacity of vitamin D binding protein. Increased public awareness of vitamin D–related health benefits might increase the risk of VDT due to self-administration of vitamin D in doses higher then recommended for age and body weight or even higher than the established upper limit intake values. Consequently, the incidence of hypercalcemia due to hypervitaminosis D might increase.

## Introduction

Vitamin D is an important prohormone that plays a vital role in maintaining healthy bones and calcium levels. Vitamin D deficiency leads to hypocalcemia and defects in bone mineralization. Vitamin D deficiency, as suggested in many publications, also is associated with increased risks of extraskeletal complications such as autoimmune diseases, chronic obstructive pulmonary disease, cancer, and metabolic syndrome. Vitamin D deficiency (25-hydroxyvitamin D [25(OH)D] concentration <20 ng/ml; <50 nmol/l) and insufficiency [25(OH)D concentration of 21–29 ng/ml; 52.5–72.5 nmol/l] are both prevalent, being a global problem of public health (1). Because of the growing awareness of vitamin D deficiency and related health problems, vitamin D became a popular supplement, and its use has increased markedly. An increased intake of vitamin D supplements by the general population and a growing number of prescriptions of therapeutic doses (including very high doses) without medical monitoring might result in a greater risk of exogenous hypervitaminosis D, with symptoms of hypercalcemia also known as vitamin D toxicity (VDT) (2). This article presents some problems associated with VDT due to an overdosing and explains some of the problems of hypersensitivity to vitamin D. The existing knowledge related to VDT is based on anecdotal case reports, accidental poisoning, and animal experiments. For ethical reasons, experimentally analyzing VDT in humans is impossible.

## Defining VDT and how often it occurs

VDT due to excess of vitamin D (hypervitaminosis D) is a clinical condition characterized by severe hypercalcemia that may persist for a prolonged time, leading to serious health consequences (3).

Hypervitaminosis D with hypercalcemia develops after uncontrolled use of vitamin D mega doses or vitamin D metabolites [25(OH)D, 1,25(OH)_2_D]. In some clinical conditions, hypervitaminosis D may develop as a result of using vitamin D analogs (exogenous VDT). Hypervitaminosis D with hypercalcemia may also be a manifestation of excessive production of 1,25(OH)_2_D in granulomatous disorders, in lymphomas, and during idiopathic infantile hypercalcemia (IIH) (endogenous VDT) (3).

In healthy individuals, exogenous VDT is usually caused by prolonged use (months) of vitamin D mega doses, but not by the abnormally high exposure of skin to the sun or by eating a diversified diet. The human body can regulate the quantity of previtamin D (tachysterol and lumisterol) produced in the skin by ultraviolet-B radiation. A diversified diet typically does not provide large amounts of vitamin D, and the fortification of food products with vitamin D is modest (4). Exogenous VDT due to vitamin D overdosing is diagnosed by markedly elevated 25(OH)D concentrations (>150 ng/ml) accompanied by severe hypercalcemia and hypercalciuria and by very low or undetectable parathyroid hormone (PTH) activity (4). Hypercalciuria and hypercalcemia are the first measurable manifestations of VDT. The1,25(OH)_2_D concentration in patients with VDT may be within the reference range, slightly increased or reduced (less frequently) when an increased level of calcium in serum suppresses PTH activity. 1,25(OH)_2_D is down regulated both by the inhibition of 1α-hydroxylase activity and by the enhancement of 24-hydroxylase activity (3).

Exogenous VDT may develop in patients taking excessive amounts of 1α,25(OH)_2_D or other 1α-hydroxylated vitamin D analogs [1α(OH)D], such as paricalcitol and doxercalciferol, used to treat hypocalcemic disorders, including hypoparathyroidism, pseudohypoparathyroidism, osteomalacia, and end-stage renal failure. In those cases, hypercalcemia is an adverse effect of treatment with use of a pharmacological vitamin D agent, not related to 25(OH)D concentration, and the 1,25(OH)_2_D concentration value is elevated (3, 5).

The increased risk of endogenous VDT is a serious clinical issue in granuloma-forming disorders and in lymphomas as well as in patients with IIH. In those disorders, patients are hypersensitive to vitamin D, and elevated 1,25(OH)_2_D concentration with hypercalcemia may develop after vitamin D supplementation or from dietary products containing increased amounts of vitamin D or even after uncontrolled sunbathing (3). Patients with Williams–Beuren syndrome also need attention for hypersensitivity to vitamin D; however, both 25(OH)D and 1,25(OH)_2_D concentration values in that disease may be either normal or elevated, and the pathophysiological explanation is often unclear. In granulomatous diseases such as sarcoidosis, tuberculosis, leprosy, fungal diseases, infantile subcutaneous fat necrosis, giant cell polymyositis, and berylliosis, endogenous VDT is related to the abnormal extrarenal synthesis of 1,25(OH)_2_D by activated macrophages (3, 6). In lymphomas, the etiology of VDT is multiple, heterogeneous, and still not fully recognized (7). In IIH, a dysfunction of 24-hydroxylase (CYP24A1) activity, an enzyme responsible for degradation of both 25(OH)D and 1,25(OH)_2_D, results in uncontrolled severe hypercalcemia and related consequences (8). IIH may be revealed in early childhood or may persist undiagnosed into adulthood (9). Another recently discovered cause of IIH involves a defect in *SLC34A1*, the gene coding for the sodium-phosphate cotransporter (NaPi-IIA) in the kidney; hypercalcemia is the indirect manifestation of the downregulation of FGF-23 (10). In endogenous VDT, hypercalcemia is related to increased 1,25(OH)_2_D concentration; in contrast, in VDT due to an overdose of vitamin D (exogenous VDT), hypercalcemia is a consequence of high 25(OH)D concentration (5).

The prevalence of VDT is unknown. As a result of increased intake of vitamin D–containing supplements and the recent information regarding prevalence of the CYP24A1 mutation (8–10) in the general population (estimated to occur in1 of 33,000 births) (11), the incidence of VDT may well increase.

In the past, exogenous VDT was considered a rare adverse effect associated primarily with food fortification. From the1930s through the 1950s, public health officials in the United States and the United Kingdom recommended routine fortification of milk and other foods with vitamin D (4). That policy was implemented initially as an effective public health strategy to prevent nutritional rickets in children and then as an intervention to improve the general health of the population (4).

In the 1940s, massive doses of vitamin D (200,000–300,000 IU/day) were considered an effective treatment strategy for chronic illnesses as diverse as tuberculosis and rheumatoid arthritis. Because hypercalcemia was observed in some patients thus treated, individual doctors discontinued the massive doses and the symptoms of VDT disappeared after a few months (4, 12). However, those clinical observations alerted physicians to the possibility of VDT, and the practice of administering massive doses of vitamin D was later discontinued nationally. Those observations, however, did not influence fortification of foods and other products with vitamin D, which persisted through the 1950s (4). In the 1950s, several cases of infants with facial abnormalities, supravalvular aortic stenosis, mental retardation, and hypercalcemia were reported mainly in the United Kingdom. That was followed by additional reports of hypercalcemia in some infants in the United Kingdom as well as in other European countries (13).

The Royal College of Physicians and the British Pediatric Association related that unexpected and unexplained increased incidence of hypercalcemia to the excessive intakes of vitamin D from various foods fortified with vitamin D. (At the time, no reliable assessment for measuring vitamin D was available, and no reliable estimates for dietary intake of vitamin D existed). The Royal College of Physicians failed to provide strong evidence for that phenomenon (they based their conclusion predominantly on literature in which pregnant rodents receiving high doses of vitamin D delivered pups with dysmorphic features, aortic stenosis, and hypercalcemia). The British Pediatric Association documented hypercalcemia only in isolated cases of infants who had approximate daily vitamin D intakes of 1,500–1,725 IU. Therefore, the U.K. government strictly regulated vitamin D food fortification and vitamin D supplements to the general public (4, 13). However, in retrospect, hypercalcemia probably resulted from hypersensitivity to vitamin D in infants suffering from Williams–Beuren syndrome and sarcoidosis (4). Nonetheless, in a substantial number of those cases, hypercalcemia was probably due to an excessive daily intake of vitamin D. Later observations of VDT came from the United States, where hypervitaminosis D in eight patients was associated with drinking vitamin D–fortified milk. An analysis of the milk produced at a local dairy revealed excessive vitamin D fortification of up to 232,565 IU per quart instead of the standard 400 IU per quart (14). As a result of that incident, local government agencies around the world prohibited the fortification of milk and alerted physicians to the potential of VDT—a concern that persists to this day (14).

In statements released over the last decade, the Institute of Medicine (IOM) (15) and the Endocrine Society (14) have both concluded that acute VDT is extremely rare in the literature, that serum 25(OH)D concentrations must exceed 150 ng/ml (375 nmol/l), and that other factors, such as calcium intake, may affect the risk of developing hypercalcemia and VDT. Regardless of additional risk factors for VDT, many studies provided evidence that vitamin D is probably one of the least toxic fat-soluble vitamins, much less toxic than vitamin A (4). Dudenkov et al. (2) researched more than 20,000 serum 25(OH)D measurements performed at the Mayo Clinic from 2002 to 2011 to determine the prevalence of VDT, demonstrated by the presence of hypercalcemia. The number of individuals with a serum 25(OH)D concentration >50 ng/ml (>75 nmol/l) had increased by 20 times during that period. However, relatively high 25(OH)D concentrations coincided with a normal serum calcium concentration. Only one patient, with a25(OH)D concentration of 364 ng/ml (910 nmol/l), was diagnosed with hypercalcemia. Pietras et al. (16) reported that healthy adults in a clinical setting, receiving 50,000 IU of vitamin D_2_ once every 2 weeks (equivalent to approximately 3,300 IU/day) for up to 6 years, maintained 25(OH)D concentrations of 40–60 ng/ml (100–150 nmol/l) without any evidence of VDT. Those findings were consistent with the observation by Ekwaru et al. (17) that Canadian adults who ingested up to 20,000 IU of vitamin D_3_ per day had a significant increase of 25(OH)D concentrations, up to 60 ng/ml (150 nmol/l), but without any evidence of toxicity.

## The process of acute VDT

VDT resulting from excessive use of vitamin D is characterized by hypercalciuria, hypercalcemia, elevated 25(OH)D >150 ng/ml (>375 nmol/l), and usually normal or slightly increased 1,25(OH)_2_D concentration.

Ten years ago, Jones (18) suggested three major hypotheses about the mechanism of VDT. All three involve increased concentrations of a vitamin D metabolite reaching the vitamin D receptor (VDR) in the nucleus of target cells and causing exaggerated gene expression. The three hypotheses to explain VDT are as follows:

1. Toxicity is mediated by increased serum concentrations of the active hormonal form, 1,25(OH)_2_D, which lead to its increased intracellular concentration. That hypothesis is not strongly supported. Only one study, Selby et al. (19) reported elevated 1,25(OH)_2_D concentration values at VDT. Many other studies revealed that 1,25(OH)_2_D concentrations were normal or only slightly elevated.
2. 1,25(OH)_2_D has a low affinity for vitamin D binding protein (VDBP) (20) and a high affinity for VDRs, making it an important ligand with access to the transcriptional signal transduction machinery. In hypervitaminosis D, the concentrations of various vitamin D metabolites, especially 25(OH)D, are markedly increased, saturating the binding capacity of VDBP and in turn enabling other vitamin D metabolites to enter the cell nucleus. Among the various vitamin D metabolites, 25(OH)D in higher concentrations (a dose-dependent effect) has the strongest affinity for VDRs, so that particular metabolite at its high serum concentrations stimulates transcription by itself (20, 21).
3. Vitamin D intake raises the concentration of vitamin D itself and increases concentrations of many other vitamin D metabolites, especially 25(OH)D. In hypervitaminosis D, the concentrations of vitamin D metabolites, such as vitamin D, 25(OH)D, 24,25(OH)_2_D, 25,26(OH)_2_D, and 25(OH)D-26,23-lactone, increase significantly (22). Abnormally increased concentrations of vitamin D metabolites exceed the VDBP binding capacity and cause a release of free 1,25(OH)_2_D; the latter active metabolite enters the target cells by diffusion and acts through the VDR.

Of those three hypotheses, abnormally high 25(OH)D and free 1,25(OH)_2_D concentrations are the most credible, although even that concept remains unproven (18, 20).

On the basis of various *in vitro* and *in vivo* studies using animal models, the mechanism of VDT suggested in hypothesis 3 seems unlikely. For example, in one study, a CYP27B1-knockout mouse lacking1α-hydroxylase and unable to synthesize 1,25(OH)_2_D still suffered from VDT when exposed to doses of vitamin D similar to those given to wild-type controls (23). Thus, the literature favors the concept that VDT involves mechanism 2 and, consequently, that serum 25(OH)D concentration represents an accurate biomarker of the risk of VDT (24).

## Signs and symptoms of VDT

The clinical manifestations of VDT are varied but are related primarily to hypercalcemia (3, 5).

Symptoms of VDT may be similar to those of other hypercalcemic states and include neuropsychiatric manifestations, such as difficulty in concentration, confusion, apathy, drowsiness, depression, psychosis, and in extreme cases, a stupor and coma. The gastrointestinal symptoms of VDT include recurrent vomiting, abdominal pain, polydipsia, anorexia, constipation, peptic ulcers, and pancreatitis. The cardiovascular manifestations of VDT include hypertension, shortened QT interval, ST segment elevation, and bradyarrhythmias with first-degree heart block on the electrocardiogram. The renal symptoms include hypercalciuria as the earliest sign, polyuria, polydipsia, dehydration, nephrocalcinosis, and renal failure. Other symptoms of VDT caused by hypercalcemia include band keratopathy, hearing loss, and painful periarticular calcinosis (25, 26).

## Diagnosis of VDT

The diagnosis of VDT can be determined clinically. An early diagnosis of VDT requires a detailed clinical and drug history. VDT in most patients is the result of excessive dosages or too-frequent dosing intervals of vitamin D administered for osteoporosis, hypoparathyroidism, hypophosphatemia, osteomalacia, or renal osteodystrophy. Because of vitamin D's current popularity as a treatment agent for many diseases, vitamin D supplementation (including use of therapeutic doses) has become predominant in otherwise healthy individuals. General practitioners should be attentive to the symptoms of VDT in patients who have supplemented with therapeutic vitamin D doses or its metabolites. When hypercalcemia develops, patients with granulomatous diseases or lymphoma have a pervasive active disease. In those cases, the diagnosis of VDT is apparent on examination (3, 5).

Laboratory findings (other than hypercalcemia) inpatients with symptomatic exogenous VDT related to overdosing of vitamin D or 25(OH)D show suppressed PTH (intact), 25(OH)D concentration>150 ng/ml (>375 nmol/l), and normal or increased values of 1,25(OH)_2_D concentration.

Exogenous VDT, as an adverse result of therapy with use of active vitamin D metabolite [both 1,25(OH)_2_D and 1α-OHD], is characterized by laboratory findings of suppressed PTH (intact), elevated 1,25(OH)_2_D concentration, and decreased or normal 25(OH)D concentration values.

Endogenous active metabolite intoxication due to coexisting granulomatous diseases or lymphoma may be characterized by suppressed PTH (intact), decreased or normal 25(OH)D concentration, and elevated 1,25(OH)_2_D.

In a hypercalcemic patient, hyperphosphatemia suggests VDT, whereas hypophosphatemia suggests primary hyperparathyroidism. The latter condition is further characterized by increased PTH activity and increased 1,25(OH)_2_D concentration but normal 25(OH)D concentration (3, 23).

## Treatment of acute VDT

Any one of vitamin D's three forms [vitamin D, 25(OH)D, or 1,25(OH)_2_D] may lead to VDT. Toxicity from vitamin D_2_ or D_3_ is harder to manage than toxicity due to vitamin D's metabolites [25(OH)D or 1,25(OH)_2_D]. That is partly due to the long half-life in the body because of vitamin D's high lipid solubility in the liver, muscles, and fat tissues and the corresponding large storage capacity (18–22).

Thus, hypercalcemia due to a vitamin D overdose theoretically can last up to 18 months after the administration of vitamin D is discontinued. That is because of the slow release of the stored vitamin D from fat deposits. However, the half-lives of 25(OH)D and 1,25(OH)_2_D in the body are much shorter, at 15 days and 15 h, respectively. Therefore, an overdose of 25(OH)D may persist for weeks, whereas that related to 1,25(OH)_2_D lasts only a few days (18, 22).

Treatment of VDT consists of first- and the second-line treatment strategies (3, 25, 27). First-line treatment includes the following:

1. Discontinuation of vitamin D supplementation and the reduction of dietary calcium intake. Patients with granulomatous diseases, lymphoma, and IIH are recommended to avoid exposure to sunlight and other ultraviolet-B light sources.
2. The administration of isotonic sodium chloride solution to correct dehydration and restore kidney function is recommended. Loop diuretics can be added once the volume is restored and maintained. In cases of prolonged sodium chloride and loop diuretic therapy, replacing lost sodium, potassium, and chloride is important.
3. Therapy with glucocorticoids (GS) will decrease plasma calcium levels by reducing intestinal calcium absorption by decreasing transcellular active transport processes and increasing urinary excretion of calcium. Furthermore, GS therapy changes the hepatic vitamin D metabolism to favor synthesizing inactive metabolites. Although that treatment is efficient (serum calcium levels usually return to normal over several days with GS at doses of 100 mg/day of hydrocortisone or equivalent), the chronic use of systemic (oral or parenteral) GS therapy is unfortunately associated with common adverse events including secondary osteoporosis, osteonecrosis, and muscle weakness.
4. Antiresorptive therapy with use of calcitonin (CT), bisphosphonates (BS), or both can be useful in severe cases in which hypercalcemia is the result of increased osteoclastic bone resorption due to 1,25(OH)_2_D's direct effect on bone tissue. The response to CT and BS is very different. CT works rapidly, but tachyphylaxis occurs after several days. BS work within a few days, but the effect persists long term. In fact, according to some reports, BS (including oral ones) are the most effective treatment for VDT, at least in children. Clinically, knowing whether increased osteoclastic bone resorption occurs is impossible, although one would assume it to be the case in the presence of significant hypercalcemia. Therefore, use of those compounds cannot be restricted to conditions of increased osteoclastic bone resorption.

Second-line treatments of VDT include the following:

5. Phenobarbital can be a useful treatment for VDT by decreasing 25(OH)D concentrations through induction of the hepatic microsomal enzyme (28).
6. Ketoconazole non-specifically decreases 1,25(OH)_2_D production by activated mononuclear cells by inhibiting cytochrome P450, CYP27B1, but long-term use is not recommended because it blocks many other important CYPs (29).
7. Aminoquinolines (chloroquine, hydrochloroquine) decrease 1,25(OH)_2_D production by activated mononuclear cells through an unknown mechanism in granulomatous diseases (30).
8. Specific inhibitors of CYP27B1 (1α-hydroxylase) have been developed that might find utility in specifically blocking the production of 1,25(OH)_2_D without interfering with other cytochrome P450–containing enzymes (31).
9. The induction of the non-specific liver cytochrome P450 enzymes, including CYP3A4, by drugs such as rifampin results in an alternative catabolic fate from the 24-hydroxylation pathway for vitamin D metabolites and allows for the non-specific breakdown of excess 1,25(OH)_2_D in IIH patients (32).

## Possible toxicity of moderate intakes of vitamin D for extended periods

The IOM Report in 2011 not only discussed the upper limits (ULs) for vitamin D intake on the basis of the acute, short-term administration of high-dose vitamin D preparations for limited periods but also emphasized chronic administration of vitamin D over years of supplementation. Acute toxicity would be caused by doses of vitamin D probably in excess of 10,000 IU/day, which result in serum 25(OH)D concentrations >150 ng/ml (>375 nmol/l). That level is clearly more than the IOM-recommended UL of 4,000 IU/day. Potential chronic toxicity would result from administration of doses above 4,000 IU/day for extended periods, possibly for years, that cause serum 25(OH)D concentrations in the 50–150 ng/ml (125–375 nmol/l) range (15).

The IOM cited several association studies that suggest possible deleterious effects of serum 25(OH)D concentrations above 50 ng/ml. Those effects include all-cause mortality, an incidence of certain cancers (breast, pancreatic, and prostate), and falls and fractures. All-cause mortality follows an inverse J curve so that risk of death appears to increase in patients with 25(OH)D concentration values above 30 ng/ml (>75 nmol/l). However, in a recent paper (33), Durazo-Arvizu and colleagues reanalyzed those findings on the basis of standardized 25(OH)D assay results and concluded that the uptick in the reverse J curve is an artifact eliminated at high 25(OH)D values.

In a controversial study, elderly women who received an annual single high dose of vitamin D (500,000 IU) had higher rates of fractures and falls than women in the control group, who received a placebo (34). Though serum 25(OH)D was not measured in the treated group, a sub-study reported that serum 25(OH)D was 48 ng/ml (120 nmol/l) 1 month after dosing. In a more recent study, Bischoff-Ferrari et al. (35) reported a higher risk of falls in men and women older than70 years, who were given 60,000 IU/month, than in control groups given 24,000 IU/month ± 300 μg of 25(OH)D_3_/month over 1 year. Serum 25(OH)D concentrations reached 40 ng/ml (100 nmol/l) in the affected group on doses of 60,000 IU/month and even higher in individuals receiving 25(OH)D_3_.

Consequently, several possible deleterious effects of chronic moderate doses of vitamin D remain unexplained. In contrast with the study of acute VDT, no plausible explanation exists for the mechanism of such deleterious effects on health with chronic VDT. Although no mechanism can yet explain those data, we must continue to question whether chronic moderate vitamin D dosing is potentially harmful.

## Summary and conclusions

Although VDT resulting in hypercalcemia is rare, it can be life-threatening if not promptly identified. Many forms of exogenous (iatrogenic) and endogenous VDT exist. The unintentional overdosing due to use of pharmaceutical products is the most frequent cause of exogenous VDT. An overview of VDT cases caused by vitamin D formulation or administration errors that resulted in excessive dosing confirmed that intoxication is extremely rare. However, VDT should always be considered as a differential diagnosis in patients with hypercalcemia (36).

In some clinical conditions, endogenous VDT is also an important clinical issue. Endogenous etiologies may develop from ectopic production of 1,25(OH)_2_D in granulomatous diseases, such as sarcoidosis and tuberculosis, or in lymphoma. Researchers have proposed many processes to account for VDT, including the inhibited activity of 24-hydroxylase or elevated activity of 1α-hydroxylase, both leading to increased concentration of the active vitamin D metabolite, the increased number of VDRs, or the saturation of the capacity of VDBP. Despite many controversies related to target 25(OH)D concentration or recommended vitamin D doses for general population, the available guidelines agree that 25(OH)D concentrations >150 ng/ml pose a significant risk of VDT and that vitamin D deficiency treatment regimens with use of high doses (higher than ULs) need regular monitoring (37).

In the general population, the awareness of vitamin D–related health benefits is growing; however, the increased consumption of vitamin D–containing supplements may predispose the general public to an increased incidence of VDT. Therefore, without medical supervision, caution is advised for people who self-administrate vitamin D at doses higher than recommended for age and body weight.

## Author contributions

All authors listed have made a substantial, direct, and intellectual contribution to the work and approved it for publication.

### Conflict of interest statement

The authors declare that the research was conducted in the absence of any commercial or financial relationships that could be construed as a potential conflict of interest. The reviewer LG and handling Editor declared their shared affiliation.
